# Twenty weeks of home-based interactive training of children with cerebral palsy improves functional abilities

**DOI:** 10.1186/s12883-015-0334-0

**Published:** 2015-05-10

**Authors:** Jakob Lorentzen, Line Z. Greve, Mette Kliim-Due, Betina Rasmussen, P. E. Bilde, Jens B. Nielsen

**Affiliations:** The Helene Elsass Center, Holmegårdsvej 28, 2900 Charlottenlund (Copenhagen), Denmark; Department of Neuroscience and Pharmacology, Panum Institute, University of Copenhagen, Copenhagen, Denmark; Department of Exercise and Nutrition, University of Copenhagen, Copenhagen, Denmark

**Keywords:** Cerebral Palsy, Children, Interactive home training

## Abstract

**Background:**

Home-based training is becoming ever more important with increasing demands on the public health systems. We investigated whether individualized and supervised interactive home-based training delivered through the internet improves functional abilities in children with cerebral palsy (CP).

**Methods:**

Thirty four children with CP (aged 9–16; mean age 10.9 ± 2.4 years) (GMFCS I-II; MACS I-II) were included in this non-randomized controlled clinical training study. 12 children (aged 7–16; mean age: 11.3+/−0.9 years) were allocated to a control group in which measurements were performed with 20 weeks interval without any intervening training. Daily activities, functional abilities of upper- and lower limbs, and balance were evaluated before, immediately after training and 12 weeks after training. The training consisted of 30 min daily home-based training for 20 weeks delivered through the internet.

**Results:**

The training group on average completed 17 min daily training for the 20 week period (total of 40 h of training). The training group showed significant improvements of daily activities (AMPS), upper limb function (AHA) and functional tests of lower limbs (sit to stand, lateral step up, half knee to standing) after 20 weeks of training. No difference was found between the test after 20 weeks of training and the test 12 weeks after training. No significance was reached for balance after training. No difference was found for any parameter for the control group.

**Conclusions:**

Interactive home training of children with CP is an efficient way to deliver training, which can enable functional motor improvements and increased activity to perform daily activities.

**Trial registration:**

ISRCTN13188513. Date of registration: 04/12/2014

## Background

Neuroplastic changes with a functional improvement can take place after training in people with lesions in the brain [1]. However, studies show that a more intensive and longer lasting training effort is needed to drive the desired neuroplastic changes than what is generally being offered people with lesions in the brain [1]. Due to practical and financial reasons home-based training is becoming ever more important for rehabilitation of a number of different conditions [2, 3]. Commercially available video-game based methods such as Wii have been shown to be efficient in promoting home-based training for adults with Cerebral Palsy (CP) [4]. However, maintaining motivation over longer periods appears to be challenging with this method. With recent developments in computer-technology, the possibility of delivering and supervising training through the internet has emerged. Such internet-based training has been tested in trials on patients with diabetes [5, 6] and rheumatoid arthritis [7] with positive results. These improvements in technology allow easier communication between the therapist and the participant, who is training, who is left with an impression that he/she has a personal coach. We recently published pilot data showing the feasibility of such training in a population of children with CP, and study protocol of other similar approaches have also been published [8, 9].

The aim of the present study was to demonstrate that interactive home-based computer training may produce important improvements in functional abilities of children with CP.

## Methods

### Subjects

Thirty four children (aged 9–16; mean age 10.9 ± 2.4 years) with the diagnosis spastic cerebral palsy (CP) (GMFCS I-II; MACS I-II) based on medical records and classification by the therapists participated in the study. The study was designed as a non-randomized controlled clinical study with a training intervention for 30 min of training pr day for 20 weeks. The effect of the training was evaluated before (pre), immediately after training (post1) and 12 weeks after training (post2). 12 children were allocated to an age-matched control group in which measurements were performed prior to (intro) and after (post) a 20 weeks interval without any intervening training. Due to different practical reasons such as illness of the children, not all children were evaluated with every test second or third time. In these cases the test results for all two or three test session were not included in the analysis for this particular test.

All children and their parents gave written informed consent to the study, which was approved by the local ethics committee of Copenhagen region (H-B-2009-017).

Table 1 summarizes demographic data and the functional abilities of the children prior to the study. Fine and Gross motor functions of the children were classified by the Manual Ability Classification System (MACS) [10] and Gross Motor Function Classification System Expanded and Revised (GMFCS-E&R) [11, 12]. MACS has been developed to classify how children with CP use their hands in during daily activities. GMFCS-E&R is a classification system that is used to describe the gross motor function of children with CP [10, 11] based on self-initiated movements with a focus on the ability to sit, stand and move [11, 12]. The classification system has 5 age groups, which each have 5 levels of function. The 5 levels are designed to ensure that the different levels reflect, as much as possible, the genuine differences in functional ability during daily living. The main focus is therefore on functional limitations and the need of walking aids rather than the quality of movement. The initial classifications of the children were made by the therapist at their first visit and the subsequent training was individualized and adjusted according to the different classes of MACS, GMFM (I-V), cognitive skills, and progression of training. The initial training was planned according to the evaluation of each child cognitive-, visual-, gross motor- and fine motor- skills at baseline. After the initial evaluation a team consisting of a physiotherapist and two occupational therapists created a program that contained generic exercises with the purpose of training cognitive-, visual- gross motor- and fine motor- skills, and exercises chosen with attention drawn towards promotion of the functions that scored lowest. A large effort was made in matching the results of the tests for each child with the optimal level of exercises for the initial training. The exercises were incremented weekly based on daily feedback regarding the training of the children. The purpose of these adjustments was to assure that the training remained challenging and motivating.

**Table 1:** Summary of the information about the children at the beginning of the training

Child number	Intervention (1) Control (2)	Gender (f)/(m)	Age (years)	GMFCS	MACS	CP type	Weight (kg)	Height (cm)
1	1	m	12	I	1	Uni	34	144
2	1	m	12	I	1	Uni right	59	161
3	1	m	14	I	2	Uni left	64	180
4	1	m	10	I	1	Uni right	40	138
5	1	f	10	I	1	Uni	35	154
6	1	f	10	I	1	Uni	32	140
7	1	m	12	I	1	Uni left	47	159
8	1	m	9	I	1	Uni left	28	130
9	1	m	9	I	1	Uni left	34	135
10	1	f	9	I	1	Uni left	37	151
11	1	f	8	I	1	Uni right	20	128
12	1	m	7	I	2	Uni left	25	130
13	1	m	7	I	2	Uni left	19	125
14	1	m	8	I	2	Uni right	31	141
15	1	f	8	I	1	Uni left	39	143
16	1	m	15	I	2	Uni left	60	164
17	1	m	14	I	2	Uni left	37	156
18	1	m	12	I	2	Uni right	40	146
19	1	f	12	II	1	Bilat	49	160
20	1	f	13	I	1	Uni right	60	155
21	1	f	11	I	1	Uni right	44	148
22	1	f	11	I	1	Uni left	31	152
23	1	m	11	I	1	Uni left	40	143
24	1	m	10	I	2	Uni right	36	146
25	1	m	11	I	2	Uni left	52	154
26	1	m	10	I	2	Uni left	43	156
27	1	m	9	I	2	Uni right	36	144
28	1	m	9	I	2	Uni right	35	142
29	1	m	10	I	1	Uni right	35	141
30	1	m	8	I	1	Uni right	21	125
31	1	f	14	I	2	Ataksi	44	159
32	1	f	13	I	2	Ataksi	50	162
33	1	m	15	I	2	Ataksi	65	181
34	1	m	16	I	1	Ataksi	65	175
	*N* = 34	f :11 m :23	Mean: 10,9 ± 0,4	I:33 II:1	*I* = 19 II = 15	Uni :29 Bilat : 1 Ataksi: 4	Mean: 40.8 ± 2.2	Mean: 149.1 ± 2.5
35	2	f	16	I	1	Uni left	40	148
36	2	f	10	I	1	Uni right	41	141
37	2	f	9	I	2	Uni right	33	139
38	2	m	7	I	1	Uni left	25	131
39	2	m	7	I	2	Bilat	22	128
40	2	m	12	I	2	Uni left	33	151
41	2	m	8	I	1	Uni left	35	139
42	2	m	11	I	1	Bilat	35	148
43	2	f	13	II	1	Bilat	32	141
44	2	m	15	I	1	Uni left	77	171
45	2	m	15	I	1	Uni right	50	168
46	2	f	12	I	1	Uni right	41	158
	N = 12	f = 5 m =7	Mean: 11.3 ± 0.9	I = 11 II = 1	I = 9 II = 3	Uni:9 Bilat:3	Mean: 38.7 ± 4.1	Mean: 146.9 ± 3.9

This table summarizes the information about the participant in this study. Columns 1–4 contain information about subject id, belonging for each subject to intervention group (1) or control group (2), gender and age. Columns 5–9 contain information about each participants score on the Gross Motor Function Classification System (GMFCS), Manual Ability Classification System (MACS), categorisation of CP, weight and height

### Measure tools

All tests were carried out at the Helene Elsass center by experienced paediatric occupational- and physiotherapists.

#### Test of daily activities

AMPS (Assessment of motor and process skills) was performed according to Fisher AG and Jones KB [13]. AMPS is a computer-based, standardized, cross-cultural assessment tool for use by occupational therapists to assess the process and motor skills of people in the age group 3–99 years. For the test, the patient selects two daily activities, which are assessed. The quality of activity is scored from the degree of exertion, efficacy, confidence and independence in 16 individual motor and 20 process skills. These are equivalent to the skills defined under ‘Activities and Participation’ in ICF [14].

#### Upper limb function test

AHA (Assisting Hand Assessment) was performed according to Krumlinde-Sundholm [15]. It is a standardized test of hand function in children aged 18 month to 12 years with unilateral functional deficits. The AHA is conducted by observing object-related actions performed with the impaired hand/arm during play with toys from the AHA test kit (suitcase).

The sum of scores may vary between 22 and 88 points, where a higher score indicates a higher ability level. The scaled scores range between 0 and 100 and is a transformation of the sum score to a percentage distribution within the scale, where 100 indicates that all test items were performed with the highest scores, and 0 means that all test items were performed with the lowest point.

### Functional strength of lower limbs

#### Sit-to-stand

The test was made according to Versuren et al. [16]. The child was placed on a stool with no back rest. Hips, knees and feet were placed in 90°. Feet were kept parallel to hip. During all measurements the child held onto a 50 cm stick to prevent support on thigh or stool. The child was requested to fully extend their hips and knees during standing before sitting again. Stand-sit was considered a full cycle, and the numbers of cycles fully carried through within 30 s were counted.

#### Lateral step-up

The children used a 20 cm stool. For each child the same height of stool was used before and after the training period. The test was performed according to Versuren et al. [16]. During the assessment the child stood next to the stool with straight hips and knees in the supporting leg. In the start of the test the leg to be tested was placed on the stool. The child was instructed to place the supporting leg (opposite to tested leg) fully on the stool with weight on legs, knees and hips straight before the supporting leg was placed on the floor again. Placement of the supporting leg on the stool and back on the floor was considered a full cycle. The number of completed cycles within 30 s was counted.

#### Half kneeling-standing

The children started the test in standing position with straight knees and hips and if necessary with one hand support. From this position the child took one large step forward to a position where one knee was touching the ground and then back to the initial position to perform one cycle. The number of completed cycles within 30 s was counted. In all cases the right leg was tested first, regardless of whether the child had right- or left sided hemiplegia or diplegia.

### Balance

#### Romberg 30 s, eyes open

The child stood without shoes and no support on a force platform (HUR balance trainer, HUR, Helsinki, Finland). Heels were held approximately 2 cm separated with an angle of 30° between the medial sides of the feet. The arms were kept relaxed at the side of the body. The platform was placed 1½ m from the wall, which had a picture with small objects that the child could focus on. The child was instructed to focus on the picture and count silently from 1 to 100. The area that the center of gravity was maintained within for 90 % of the time (C90 area), the average velocity of sway and the total trace length were registered.

### Training procedure

The training of the children took place in their own home over a 20 week period. The program delivered to the children ensured that they trained 30 min per day every day during the whole period. The training was delivered through the internet and consisted of a server-based interactive training-system using flash-technology. The system has been developed through collaboration between The Helene Elsass Center, a private software development company (Head-fitted; Århus, Denmark) and the University of Copenhagen. It has now been made commercially available through collaboration between the Helene Elsass center and the Ministry of Research under the name Mitii (Move It To Improve It; Mitii developments, Charlottenlund, Denmark). The training-system is designed to combine cognitive and motor challenges in order to train cognitive, perceptual and motor abilities at the same time. It consists of a number of training modules in which the child has to analyse visual information, solve a cognitive problem (i.e. mathematical question or similar) and respond with a motor act to objects presented on the screen (i.e. bend to pick up needle and blow up balloon with the right answer). The core of the system is that the computer program identifies the movements of the child from video images sampled from a simple web-camera attached to the computer. No additional computer-interface is thereby necessary – the child may control the computer-program by its own free movements. The motion detection is based on identification of a green band, which may be placed around the wrist, head or leg of the child.

The level of difficulty may be adjusted throughout the training period by increasing the difficulty of the perceptual (e.g. increasingly complex forms have to be correctly identified), cognitive (e.g. increasingly difficult mathematical questions) or motor challenges (e.g. child has to do more repetitions or work with higher load). This adjustment was executed by therapists weekly (PT and OT), who followed the training of the child through the internet based on daily feedback regarding the progress of the child. The therapists were in addition in contact through E-mail and Skype with the child and its parents on an at least weekly basis and thereby received further feedback regarding the progress of the training. This had the additional effect that the child (and its parents) had the impression thought that they had a ‘private’ virtual coach who supervised the training. This has been reported as one of the most motivating factors by both parents and children [8].

The different modules were combined uniquely according to the specific motor deficits of each child with special focus on training of the childrens upper-, lower limb and balance abilities. The system thus has two clear advantages over existing training programs: It is highly adjustable to the individual needs of the child and it can be incremented according to the progress of training of the individual child.

The individual Mitii training packages were made initially at the base of three categories including 3–4 modules with different themes such as basketball, mathematics or aeroplane. One category for lower limb modules, one for balance and one for upper limb modules.

#### Upper limb training modules

In this category the aim was to train the childrens ability to use their upper limbs in relation to reaching in different directions.

1. Mathematics - balloon module.
  Marker on impaired hand. Child was required to bend down to pick up needle and reach high to smash balloon with correct answer.
2. Memory.
  Marker on impaired hand. Child was required to pick up correct figures placed in different areas of the screen after memorizing a set of figures shown briefly on the screen.
3. Jigsaw puzzle moving.
  Marker on impaired hand. Child was required to catch bits of a puzzle moving up and down on the left or right side of the screen.
4. Identify correct shape.
  Marker on impaired hand. Child was required to pick up the figure among many placed in different areas of the screen that corresponded to a target figure. The correct figure would be a mirror image of the target figure.
5. Find corresponding figures.
  Marker on both hands. Child was required to grasp a figure in one side of the screen with one hand and bring it to a target in the middle of the screen, while grasping a corresponding figure in the other side of the screen and bring it to the target at the same time.
6. Jigsaw puzzle still.
  Marker on the impaired hand. The child was required to take a bit of a puzzle and place it where it fits in the big picture by dragging it from the bottom to the top.
7. Follow a figure that is moving.
  Marker on both hands. The child was required to use both arm to follow a figure moving on the screen.
8. Find the figure that is different.
  Marker on the affected hand. The child was required to find the figure that is different form the others and place his/her hand over this.

#### Lower limb training modules

The aim of this category was to increase strength in the lower limbs and improve control of movements during functional activities that include the use lower limbs.

1. Basketball.
  Marker was placed on the head. Child was required to bend down and jump up to shoot basketball.
2. Mathematics - balloon module
  Marker on hand. Child was required to bend down to pick up needle and reach high to smash balloon with correct answer.
3. UFO.
  Marker on head. Child was required to steer flying UFO through narrow corridors without hitting walls by shifting balance from side to side and moving up and down.
4. Man with umbrella.
  Marker on head. Child was required to keep umbrella over the head of a man so that he would not get wet from a rain shower, while walking from side to side and up and down on the screen.

#### Balance-oriented training modules

The aim of this category was to maintain a stable and secure position during change of limb and body postures according the feedback from the screen.

1. Aeroplane.
  Marker on head. Child was required to steer aeroplane by shifting balance from side to side.
2. UFO.
  Marker on head. Child was required to steer flying UFO through narrow corridors without hitting walls by shifting balance from side to side and moving up and down.
3. Man with umbrella.
  Marker on head. Child was required to keep umbrella over the head of a man so that he would not get wet from a rain shower, while walking from side to side and up and down on the screen.

For further information and illustrations of the system see Bilde et al. 2011 [8].

### Data analysis

Linear mixed models (fixed effect analysis) (covariance parameter - best model fit) was used to test for interaction effects between the intervention group and control group before and after the 20 weeks of training/control period and 12 weeks after training. The analyses were made in IBM SPSS 22 statistics. Post hoc test for differences between measurements before training, after the 20 week training and 12 weeks after training, and differences between the measurements of the control group were made using the Sidak method.

All data are given as population mean ± 1 SEM. The significance levels were set to *p* < 0.05.

## Results

### Training duration

The 34 children in the training group on average completed the daily 30 mins training program on 78.0 ± 36.3 days (range: 17–134 days) out of the scheduled 140 days. This corresponds to an average of 56 % in the 20 week period. However, on 128.0 ± 12.8 days (range: 91–140 days) the training was started, but not completed. This corresponds to 91 % of possible days of training. On average the children thus trained 17 min pr day for the 20 week period. This corresponds to 40 h of total training time. One of the main reasons for the difference between the actual amount of training and the aim of 140 full days were technical problems and in some cases that the child was to too tired or upset which made it difficult for the children to complete the training of the day. We found no relation between the number of days of training and the extent of improvement in any of the functional tests.

#### Subjective reports

All reports from the children and their families about their experiences were very positive. Despite some concerns during the training period about how to maintain the energy required to train intensively for 30 min every day all families reported that they found this way of training very positive and appealing.

Some exercises were reported to be boring by some children and not by other children. Also some exercises were reported too easy or too difficult. All families reported that the child showed several signs of improved activity in daily life. Most families reported that the child increased participation in daily activities at school and during leisure time. Also most families reported that the child showed signs of increased self-confidence and self-esteem.

All families reported that specific skills such as bicycling, eating and attention skills were improved during the training. Several also reported increased muscle strength and increased endurance.

#### Test of daily activities

**Fig. 1:**
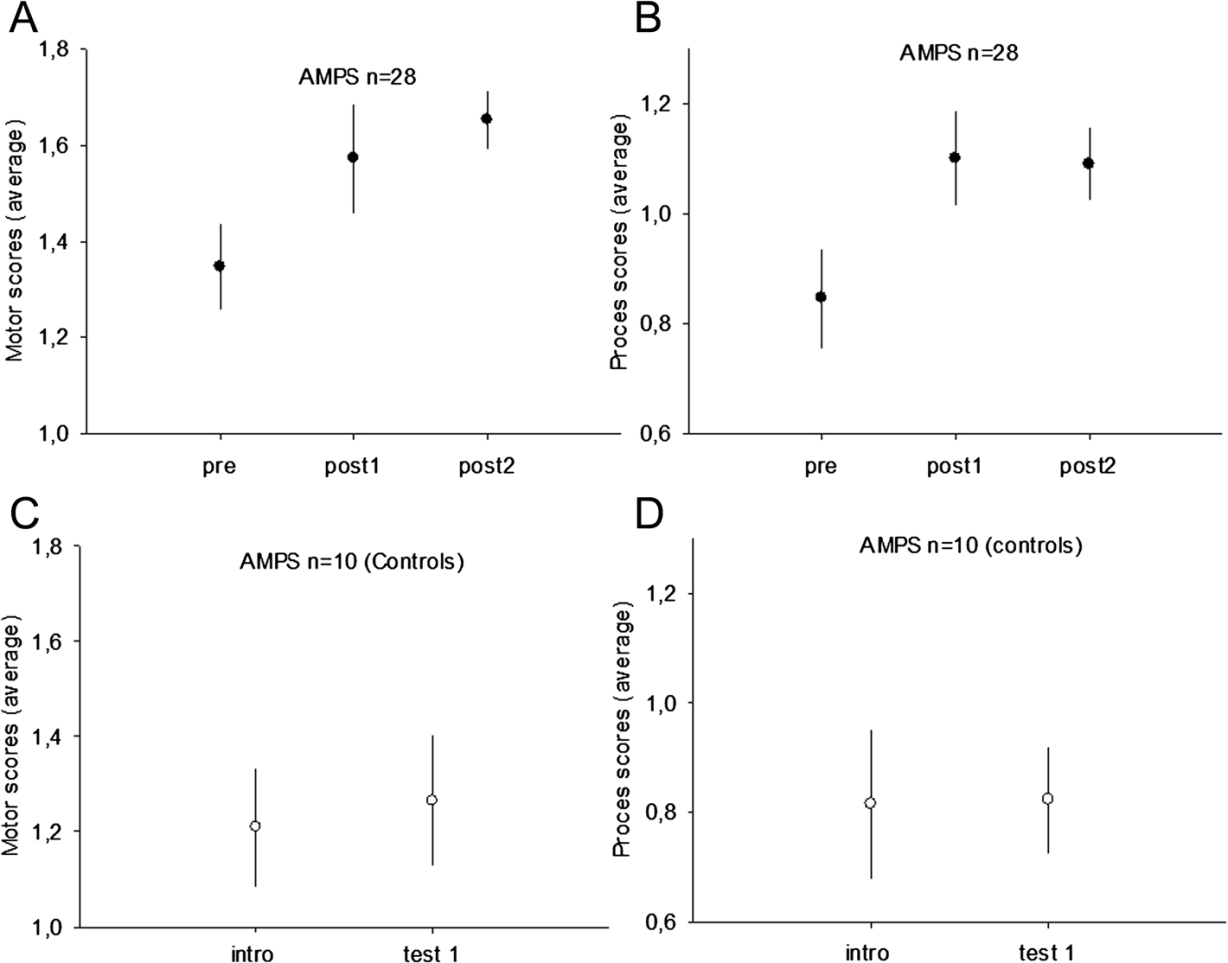
Assessment of motor and process skills (AMPS) scores are displayed for 28 children with cp in the intervention group before training (pre 1), after 20 weeks of training (post1) and 12 weeks after the training period (post 2) in (**a**, **b**). The motor scores are displayed in (**a**) and process scores in (**b**). The AMPS scores for the control group are displayed before (intro) and after (test 1) the 20 weeks period with no intervention for the motor scores (**c**) and process scores (**d**)

A significant interaction effect for the training/control group and pre training/post training was found for the AMPS test (motor: *p* = 0.049; process: *p* = 0.04). The score for motor skills was increased after the training for the 28 children who completed the training period and the AMPS test from an average of 1.34 +/− 0.09 (pre) to 1.57 +/− 0.11 (post1) (*p* < 0.001) (Fig. 1a). No significant difference was found between the test immediately after training and the test 12 weeks after training 1.57 +/− 0.11 (post1) to 1.65 +/− 0.06 (post2) (*p* = 0.84).

The process skills were significant increased after training from 0.85 +/−0.09 (pre) to 1.10 +/−0.09 (post1) (*p* < 0.001) whereas no difference was found between the test immediately after training 1.10 +/−0.09 (post1) and 12 weeks after training 1.09 +/−0.07 (post2) (*p* = 0.58) (Fig. 1b).

No significant difference was found between the two tests in the 10 children who completed the AMPS in the control group for either motor 1.21 +/− 0.12 (intro), 1.26 +/− 0.14 (*p* = 0.48) or process skills 0.82 +/−0.13 (intro), 0.82 +/−0.10 (*p* = 0.95) (Fig. 1c, d).

#### Upper limb function test

**Fig. 2:**
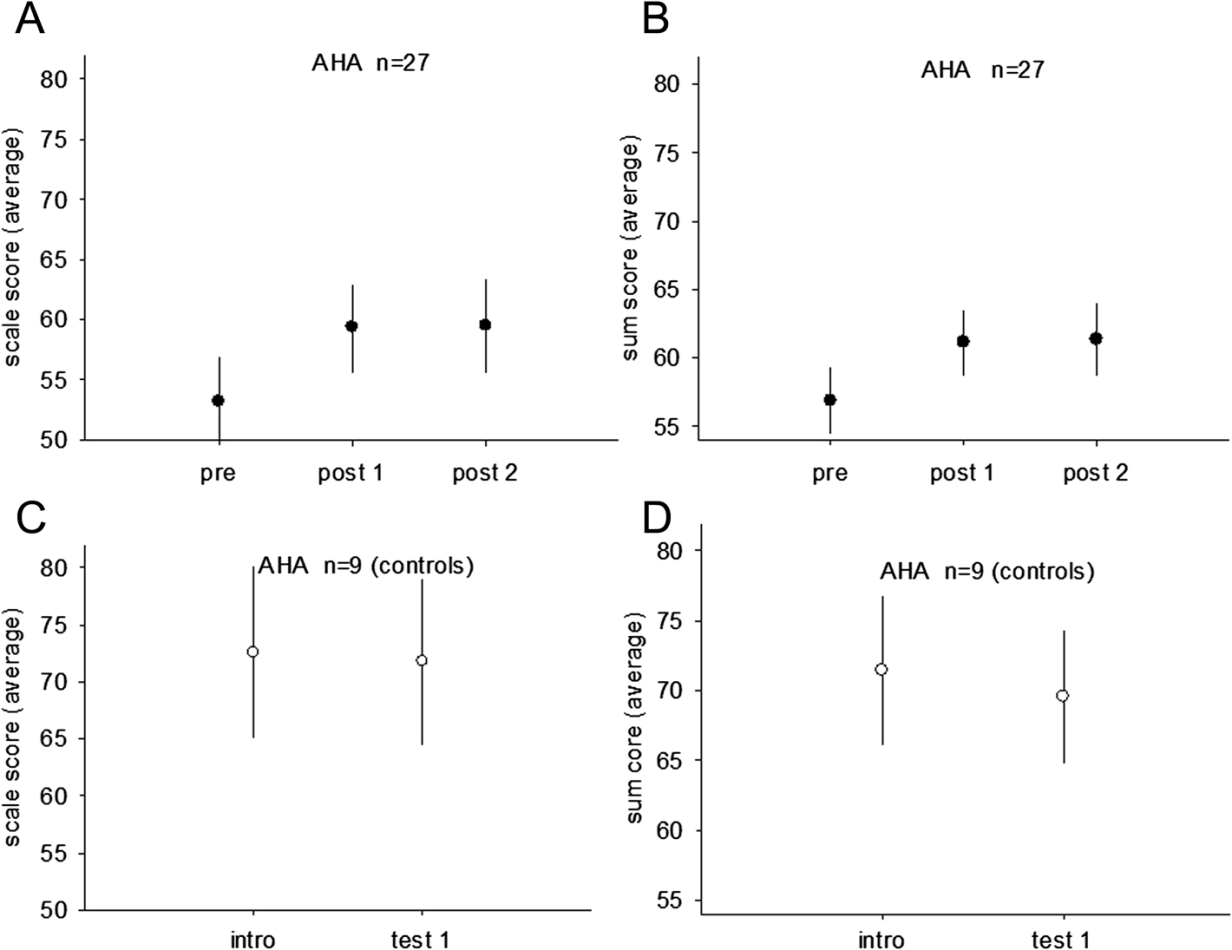
AHA (Assisting Hand Assessment) scores are displayed for 27 children with cp in the intervention group before training (pre), after 20 weeks of training (post 1) and 12 weeks after the training period (post 2) in (**a**, **b**). The scale scores are displayed in (**a**) and sum scores in (**b**). The AHA scores for the control group are displayed before (intro) and after (test 1) the 20 weeks period with no intervention for the scale scores (**c**) and sum scores (**d**)

A significant interaction effect for the training/control group and pre training/post training was found for the AHA test (scale score: *p* < 0.001; sum score: *p* < 0.001). The improvements were significant after training for the AHA scale score for the 27 children completing the tests from an average of 53.2 +/−3.7 (pre) to 59.3 +/−3.6 (post1) (*p* < 0.001). A further significant increase was found from immediately after training to 12 weeks after training 59.3 +/−3.6 (post1), 65.2 +/−2.7 (post2) (*p* = 0.03) (Fig. 2a). A significant change was also found for sum score after training from 56.9 +/−2.4 (pre) to 61.1 +/−2.4 (post1) (*p* < 0.001). No significant change was found between the tests immediately after training to 12 weeks after training 61.1 +/−2.4 (post1), 61.3 +/−2.6 (post2) (*p* = 0.80) (Fig. 2b). No change was found in the 9 control children who completed the tests for sum score 71.4 +/−5.3 (intro), 69.6+/−4.8 (test1) *p* = 1.0; scale score 72.6 +/−7.5 (intro) 71.8 +/−7.3 (test1) *p* = 0.32) (Fig. 2c, d).

#### Functional strength tests of lower limbs

**Fig. 3:**
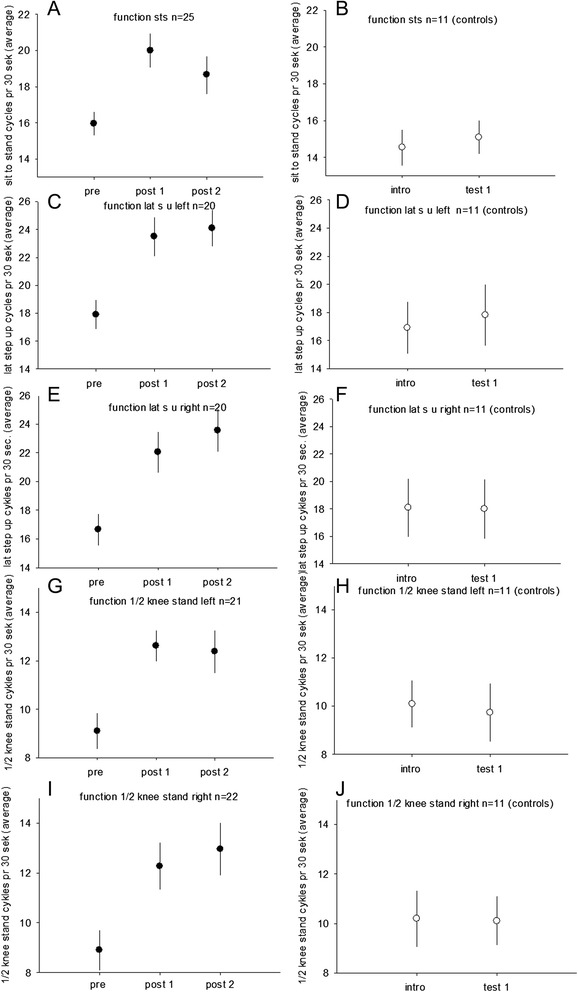
Functional strength is displayed for children with CP in the intervention group before training (pre), after 20 weeks of training (post 1) and 12 weeks after the training period (post 2). The scores for the control group are displayed before (intro) and after (test 1) the 20 weeks period with no intervention. (**a**, **b**) show the average number of stand to sit cycles for the children with CP (A, filled circles) and for the children in the control group (B, *open circles*). (**c**-**f**) show the number of lateral-step-up cycles for *left leg* (**c**, **d**) and right leg (**e**, **f**). (**g**-**j**) show the number of half kneeling-standing cycles for the *left leg* (**g**, **h**) and *right leg* (**i**, **j**)

The sit to stand test was performed in 25 children from the training group and 11 children from the control group. A significant interaction effect for the training/control group and pre training/post training was found for the STS test (scale *p* = 0.04). A significant increase in the number of sit to stand cycles performed during the tests was found after the training period from 16.1 +/− 0.7 (pre) to 19.2.0 +/− 0.9 (post1) (*p* = 0.01) (Fig. 3a). No significant change was found between the test immediately after the training and 12 weeks after training 20.0 +/− 0.9 (post1), 18.7 +/− 1.0 (post2) (*p* = 0.58).

No significant change was found for the control group 14.5 +/− 1.0 (intro) 15.1 +/− 0.9 (test1) (*p* = 0.33) (Fig. 3b).

The lateral step-up tests were performed in 20 children in the training group and in 11 control children. A significant interaction effect for the training/control group and pre training/post training was found (left leg: *p* = 0.004; right leg: *p* < 0.001). A statistically significant increase of the number of steps after training was observed for the left leg from 17.9 +/− 1.1 (pre) to 23.5 +/− 1.4 (post1) (*p* < 0.001) and right from (16.7 +/− 1.1 (pre) to 22.1 +/− 1.4 (post1) steps (*p* < 0.001) (Fig. 3c, e). No significant change was found between the test immediately after (23.5 +/− 1.4 (left); 22.1 +/− 1.4 (right) and 12 weeks after training (24.1 +/− 1.3 (left) (*p* = 0.63); 23.6 +/− 1.5 (right) (*p* = 0.17).

No significant change was found for the control group for left from (16.9 +/− 1.8 (intro) to 17.8 +/− 2.2 (test1) (*p* = 0.44) or right (18.1 +/− 2.1 (intro) to 18.0 +/− 2.0 (test1) (*p* = 0.93) (Fig. 3c, f).

The half knee-standing tests were performed in 21 children in the training group and in 11 control children. A significant interaction effect for the training/control group and pre training/post training was found (left leg: *p* = 0.03; right leg: *p* = 0.01). A statistically significant increase of the number of steps was observed after the training for left from 9.1 +/− 0.7 (pre) to 12.6 +/− 0.6 (post1) (*p* < 0.01) and right from 8.9 +/− 0.8 (pre) to 12.3 +/− 1.0 (post1) (*p* < 0.001) (Fig. 3g, i). No significant change was observed between the test immediately after training 12.6 +/− 0.7 left; 12.3 +/− 1.0 right to 12 weeks after training 12.4 +/− 0.9 left, *p* = 0.72; 13.0+/−1.1 right (*p* = 0.31). No change was observed for the control groups (left; *p* = 1.0; right; *p* = 0.89) (Fig. 3g, h, i, j).

#### Balance test

**Fig. 4:**
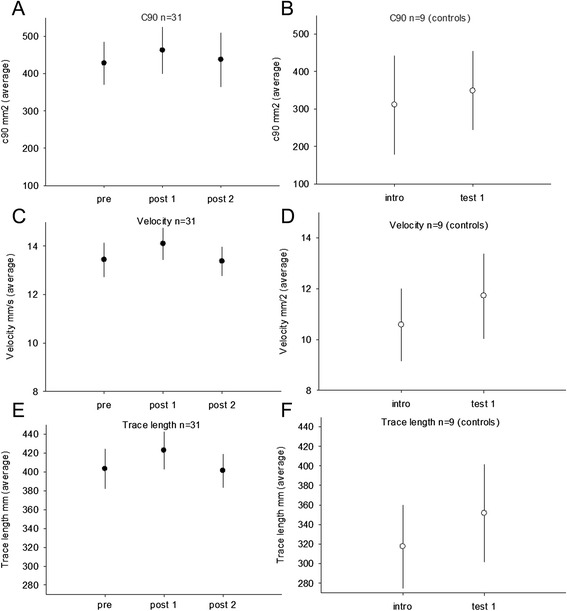
The balance parameters are displayed for children with CP in the intervention group before training (pre), after 20 weeks of training (post 1) and 12 weeks after the training period (post 2). The scores for the control group are displayed before (intro) and after (test 1) the 20 weeks period with no intervention. Filled circles indicate CP and open circles control. (**a**, **b**) show the average C90 in mm2. (**c**, **d**) show the average velocity mm/s and (**e**, **f**) show the average trace length mm

No significant interaction effect for the training/control group and pre training/post training was found for any of the Romberg tests for the 31 children who performed the training and were tested for balance or the 9 control children (C90: *p* = 0.18; vel: *p* = 0.59; trace length *p* = 0.77) (Fig. 4a-f).

The C90 found in the intervention group was 427.2 +/− 57.6 mm2 (pre) and 462.2 +/− 62.5 mm2 (post1). For the control group C90 was 310.9 +/− 131.9 mm2 (intro) and 314.6 +/− 104.9 mm2 (test1) (Fig. 4a, b). The velocity for the intervention group was 13.4 +/− 0.7 (pre) and 14.1 +/− 0.7 mm/s (post1). In the control group the velocity was 10.6 +/− 1.4 (intro) and 11.7 +/− 1.7 (test1) (Fig. 4c, d). The trace length in the intervention group was 403.1 +/− 21.3 mm (pre), 422.8 +/− 19.9 mm (post1) and in the control group 317.4 +/− 43.0 mm (intro) and 351.6 +/− 50.2 mm (test1) (*p* = 0.9) (Fig. 4e, f).

## Discussion

This study has demonstrated that clinically important functional benefits may be obtained when children with CP are motivated to train intensively daily over a prolonged period using interactive computer-based training delivered through the internet.

The children on average trained for 17 min every day for 20 weeks; i.e. a total of 40 h of training each. Unsurprisingly, with this amount of training significant improvements in a range of functional tests were observed following the intervention period, but not for a group of the children following a control period of similar duration. The effects observed following the training are therefore unlikely to be caused by the growth of the children or similar changes that are unrelated to the training. The children were not in any specific growth phase during the intervention period as compared to the control period and should on all other parameters be more optimally matched than what is normally accomplished when using a separate group of control children.

The increase in the performance of the functional lower limb tests (sit to stand, lateral step-up, half knee-standing) observed here was generally larger than that observed in randomized controlled and blinded trials involving lower limb strength and/or function training [2]. However, in these studies much less training in terms of intensity and duration was conducted during the intervention period. The improvements in upper limb function (AMPS, AHA) were also similar to other studies with similar duration of training [17] and generally larger than those reported in randomized controlled studies involving specific upper limb functional training with less duration of training [18]. The improvements found in this study are therefore in all likelihood explained by the intensity and long duration of the training. Whereas the children in our study trained every day and for an average of 40 h in total, children have only trained every second day at the most and for only a total of 16 h in published randomized controlled trials [18]. In this comparison it should also be taken into account that our children trained both upper and lower limb as well as balance and therefore did not spend all 40 h training one specific function which is the norm in most other studies [17, 18]. It should also be taken into account that our children had a variable symptomatology and suffered to a different extend from affliction of lower and upper limbs and/or balance. Some children therefore probably did not benefit from training of functions with which they had no problems prior to the training. Although the training was to some extent directed towards the functional problems of the individual child, and children with upper limb problems therefore primarily received upper limb training, they also spend a considerable time training other functions in order to ensure variability in the training sessions and thus maintain motivation. It is therefore conceivable that even larger training effects would have been observed if we had included only children with problems influencing the examined limb or function, but we chose not to do this because we also wanted to investigate if effects in multiple functional domains and not only the specifically trained domain could be recognized after training.

The large training effect obtained in the present study, as compared to other training studies in children with CP, may potentially also be related to the multi-modality of the training and possibly also that the training involved not only training of motor functions but also training of various executive and cognitive functions such as attention and working memory. Without a direct comparison with a uni-modal intervention that does not involve training of executive and cognitive functions, it is difficult to conclude whether this played a direct role for the positive training effects or not. However, there is no doubt that the children would not have finished all 20 weeks of training if not for the multi-modality and cognitive challenge of the training, and from this point of view this approach was therefore indispensable for the training effect.

It is also difficult to conclude whether the training effects were of significance for the daily life and daily functional capacity of the children. The sizes of the training effects for both the lower and upper limb were certainly of a magnitude where it has been claimed in other studies that clinical benefits must be expected [2]. The reports from the children and the parents were also generally consistent with this. In line with this it should also be noted that all the functional changes observed immediately after the training were retained at the 12 week follow up. This suggests that the training effects generated a genuine lasting lift in the functional capacity of the children.

Although several of the training modules were directed at training balance, no significant changes were observed in any of the balance tests. This is similar to what was reported for a much smaller sample of children [8]. All children did improve their performance on the actual balance tasks in the training modules (see Methods) so one possible interpretation of these negative findings is that improved balance in those tasks did not carry over to the circumstances during the balance testing. This may either be because the training effects were specific for the circumstances during the computer-training modules or that the circumstances during the balance testing were too different from the circumstances during the training to demonstrate any effect. It may be that the children were simply not challenged sufficiently during the balance tests to demonstrate the improvement in their balance. The sensitivity of the balance tests in demonstrating clinically important changes in balance has been questioned before [19].

## Conclusion

As children in our study trained every day, which is normally not the case in conventional physio- and occupational therapy, we believe that both the intensity and the volume of training achieved were much higher than what is easily achieved with other means. The Mitii training system thus offers an important step towards achieving the number of repetition of exercises, which appears necessary to drive neuroplastic changes. We consequently conclude that interactive home-based training delivered and supervised through the internet is an efficient way of providing multi-modal training of children with cerebral palsy that may lead to lasting functional benefits.
